# A large apical pseudoaneurysm of the left ventricle ruptured into the pericardium

**DOI:** 10.11604/pamj.2020.36.189.22696

**Published:** 2020-07-16

**Authors:** Ayoub Abetti, Marouane Ouazzani Ibrahimi

**Affiliations:** 1 Department of Cardiovascular Surgery, Arnaud de Villeneuve Hospital, University Hospital of Montpellier, Montpellier, France,; 2 Department of Cardiovascular Surgery, Mohammed VI University Medical Center, Mohammed First University, Oujda, Morocco

**Keywords:** Pseudoaneurysm, left ventricle, DOR technique

## Image in medicine

This is the case of a 78 years old patient with a history of acute coronary syndrome complicated by rupture of the left ventricle. The ventricular wall defect was closed by tachosyl heamostatic patchs and gluing. Two months after the occurrence of the acute coronary syndrom, computed tomography detected a huge apical pseudoaneurysm of the left ventricle ruptured into the pericardium (A). The patient underwent median sternotomy and cardiopulmonary bypass associated with a moderate hypothermia at 28°C, then, we performed the DOR technique for the cure of the pseudoaneurysm (B), using a Gore Tex patch and Teflon felt support (C,D).

**Figure 1:**
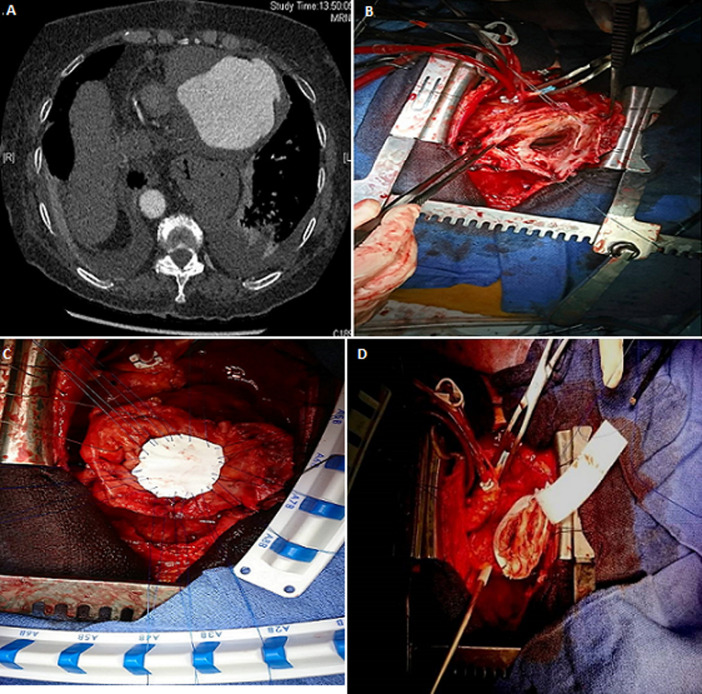
A) computed tomography showing a huge apical pseudoaneurysm of the left ventricle with extravasation of the radiocontrast agent in the pericardium; B) a perioperative view showing the apical pseudoaneurysm of the left ventricle; C) a perioperative view showing the closure of the defect by a Gore Tex patch; D) a perioperative view showing the closure of the remaining re-entries by Teflon felt support

